# Complicating the dominant morality discourse: mothers and fathers’ constructions of substance use during pregnancy and early parenthood

**DOI:** 10.1186/s12939-015-0206-7

**Published:** 2015-08-25

**Authors:** Cecilia Benoit, Samantha Magnus, Rachel Phillips, Lenora Marcellus, Sinéad Charbonneau

**Affiliations:** Centre for Addictions Research of BC and Department of Sociology, University of Victoria, PO Box 3050, Victoria, V8W3P5 BC Canada; School of Public Health and Social Policy, University of Victoria, PO Box 1700, Victoria, V8W2Y2 BC Canada; Centre for Addictions Research of BC, Centre for Addictions Research of BC, Victoria, Canada; School of Nursing, University of Victoria, PO Box 1700, Victoria, V8W-2Y2 BC Canada; Faculty of Law, University of Toronto, #1- 328 Arlington Ave, Toronto, M6C2Z9 ON Canada

**Keywords:** Substance use, Pregnancy, Parenthood, Social determinants of health, Abstience, Autonomy, Harm reduction

## Abstract

**Introduction:**

Consumption of substances is a highly controversial behaviour, with those who do so commonly viewed as deviants, even criminals, or else as out of control addicts. In other work we showed that the use of substances by women who are pregnant or have recently become parents was mainly viewed by health and social care providers as morally wrong. Problematic substance use was framed through the narrow lens of gendered responsibilisation, resulting in women being seen primarily as foetal incubators and primary caregivers of infants.

**Methods:**

In this follow-up paper we examine descriptive and qualitative data from a convenience sample of biological mothers and fathers (*N* = 34) recruited as part of a larger mixed methods study of the development and early implementation of an integrated primary maternity care program. We present a description of the participants’ backgrounds, family circumstances, health status, and perception of drug-related stigma. This is succeeded by a thematic analysis of their personal views on substance use during both pregnancy and the transition to parenthood.

**Results:**

Our results show that while many mothers and fathers hold abstinence as the ideal during pregnancy and early parenting, they simultaneously recognize the autonomy of women to judge substance use risk for themselves. Participants also call attention to social structural factors that increase/decrease harms associated with such substance use, and present an embodied knowledge of substance use based on their tacit knowledge of wellness and what causes harm.

**Conclusions:**

While these two main discourses brought forward by parents concerning the ideal of abstinence and the autonomy of women are not always reconcilable and are partially a reflection of the dissonance between dominant moral codes regarding motherhood and the lived experiences of people who use substances, service providers who are attuned to these competing discourses are likely to be more effective in their delivery of health and social services for vulnerable families. More holistic and nuanced perspectives of health, substance use, and parenting may generate ethical decision-making practice frameworks that guide providers in meeting and supporting the efforts of mothers and fathers to achieve well-being within their own definitions of problematic substance use.

## Introduction

Substance use during pregnancy and early parenthood is seen in Canada and most other countries as a significant public health problem. Infants with exposure to substances in utero are believed to face multiple health and social challenges, including Fetal Alcohol Spectrum Disorder (FASD), neonatal withdrawal, and ultimately, higher incidence of placement in foster care and greater risks of perceived and actual child abuse [1, 2]. In Canada it is thought that anywhere between 1 in 500 to 1 in 3000 children are born with Fetal Alcohol Syndrome (FAS) [3]. Incidence of more subtle FASD or alcohol-related birth defects (ARBD) are thought to be higher [4–6], but stigmatization of maternal alcohol consumption and ambiguity in diagnostic criteria [7] are thought to lead both to under-reporting and over-reporting, respectively. The Canadian Institute for Health Information (CIHI) reported that in Canada in 2003–2004, 171 newborns were diagnosed with neonatal abstinence syndrome (NAS), with an increase to 654 reported cases in 2010–2011 [8]. This represents in large part an increase in attention and awareness on the part of health professionals.

Yet the evidence of what is harmful about substance use during the reproductive period is far from clear. In the case of infants exposed to maternal cocaine while in utero, early reports predicted a dire future and the myth of the “crack baby” began [9]. However, more recent studies suggest that for many drugs, including cocaine, evidence on specific physiological and developmental effects of prenatal substance exposure is still inconclusive and that social-environmental contexts play a much larger role than previously thought [10–12]. Gender is a fundamental determinant of health for this population, intersecting with other key determinants (such as social class, race, and age), as it influences access to important life resources, including employment, education, childcare, safe neighbourhoods, and health services [13–15]. These factors are necessary considerations when contextualising substance use during the reproductive period and impact perceptions of pregnancy and parenting. For example, surveillance of maternal substance use intersects with class and racial discrimination, a reality that explains the problematizing of Indigenous women within discussions of parental substance use [16]. The stigma associated with maternal substance use also engenders a host of social, material and psychological marginalisations that have adverse consequences for both the mother and her child [17].

Stigmatizing attitudes have likewise been shown to pose a significant barrier for women accessing services and receiving adequate care [18, 19]. Radcliffe (2011) found that once pregnant women received the label of “substance user”, they were often seen by their medical practitioners to be disrespectful, incompetent parents, and untrustworthy [20]. Fear of losing their children through Child Protection Services (CPS) interventions and apprehensions is another major barrier that stops mothers who use substances from reaching out for support, particularly when there are other compounding marginalisations such as lack of housing and racism [17, 21, 22]. Issues such as non-disclosure, stigma, harm reduction, and trauma have become prominent in recent literature and researchers are increasingly developing guidelines for health practitioners to use supportive and nonjudgmental practices as well as sensitive and equitable screening tools [2, 23, 24].

In previous work we analyzed interviews with health and social service providers associated with a harm reduction initiative for pregnant and early parenting women known as the HerWay Home program situated in Victoria, BC. HerWay Home is informed by a harm reduction philosophy and a social determinant of health framework [25] that recognizes the importance of providing services to help reduce the risk environments of vulnerable families [26]. Most of the service providers associated with HerWay Home that we interviewed regarded any substance use by women during the reproductive period as morally wrong and essentially insupportable. This notion of problematic substance use was framed within an individualized gendered responsibilisation perspective that depicts women as foetal incubators and primary caregivers [27].

One of the shortcomings in the research literature, including our earlier study, is that the voices of the parents themselves are absent [28–31]. In this paper we begin to fill this gap by capturing the perceptions of clients who have used or currently use substances and are eligible to access HerWay Home services. Most of the participants we interviewed were mothers; however, we will also share the perspectives of a small number of biological fathers. We focus, in particular, on parents’ individual conceptualisations of substance use during pregnancy and the transition to parenthood. We first present the methods used for the study, and then describe our sample, shedding light on the socio-economic context of their lives and the structural barriers they face that negatively impact their health and access to services. We then present our qualitative results based on parents’ answers to the question: “How do you define problematic substance use during pregnancy and early parenting?”

## Methods

The data presented below were gathered as part of a mixed-method study entitled, *Treatment and Prevention of Illicit Substance Use among Pregnant and Early Parenting Women*, which was funded by the Canadian Institute of Health Research and received ethical review from the Human Research Ethics Board at the University of Victoria, Canada. While the purpose of broader study was to inform client harm reduction services offered through HerWay Home, the specific focus of the analysis presented here is to shed light on the views of structurally marginalized expectant and recent parents regarding substance use during pregnancy and early parenting.

Five members of the research team conducted 34 in-person interviews with participants (26 self- identified women; 8 self-identified as men) who said they were directly or indirectly affected by substance use, had low income or insecure housing, and who had been pregnant or had a child (or had a pregnant partner) in the last 12 months. Participants were recruited through postering in health and social service sites and community centres frequented by families, by referrals from the HerWay Home service provider network, and by snowballing. Participants gave their permission to have their interviews recorded. The audio-recorded interviews averaged approximately two hours in duration. A modest honorarium and public transit passes were provided to facilitate participants’ access to interviews. Answers to the closed-ended questions were entered into the statistical package SPSS and the open-ended answers were transcribed verbatim. The thematic groupings in the qualitative findings were derived by a series of steps. First, using NVIVO software, the second author read and re-read the data to identify a coding strategy which would allow for a description of the range of ideas presented about problematic substance use. Following this, the third author (who had conducted many of the interviews) independently analysed the data to assess the validity and reliability of the initial coding and to provide feedback on presentation of the most salient themes. The results of the initial coding strategy were reviewed with the first author to develop a theoretical interpretation of the data. After identifying the range, as well as the most salient ideas, raised by participants, a final coding step involved checking the coding of data to ensure that the coding strategy had been reliably executed across the interviews.

## Results

### Participants’ profiles

The majority of participants identified as women (26, 76 %) and half (17, 50 %) identified as of Aboriginal background (Status and non-Status Indian or Métis). The mean age of the participants was 29 years and nearly half were under 25 (only participants aged 19 and over were recruited). A substantial minority had not finished high school, and most participants lived on very low incomes. Their median personal annual income of $10,700 and median household annual income of $12,000 were substantially lower than the national median personal income ($30,180) and Canadian median household income ($61,072) [32] Adding to economic vulnerability, over two-thirds (23, 70 %) were currently recipients of social assistance, and only three participants reported having some form of employment at the time of the interview (Table 1).

**Table 1:** Demographic data (*N* = 34)

Demographic Profile (34 participants)	
Gender and Age	
Female	26 (76 %)
Male	8 (24 %)
Age (mean)	29 years
Under 25	15 (44 %)
Ethnicity	
White	15 (44 %)
Aboriginal	17 (50 %)
Visible minority (non-Aboriginal)	2 (6 %)
Education	
Completed grade twelve	19 (59 %)
Currently enrolled in school	6 (19 %)
Employment and Income	
Personal annual income (median)	$ 10,700
Household annual income (median)	$ 12,000
Currently employed	3 (9 %)
Current recipient of income assistance	23 (70 %)
Applied for income assistance and were turned down in the last 12 months	7 (21 %)

The majority of participants had at least one child already (29, 85 %), and of these nearly half (14, 48 %) had at least one child living outside their care. While the majority of participants said they had secure housing (25, 74 %), one-quarter stated their housing was somewhat insecure or very insecure (9, 26 %); of the latter, a few (5, 15 %) reported being “without housing”. Half of participants reported living with partners (17, 50 %), though three of these shared living only part-time.

Participants reported poorer health than other Canadians. Near half (14, 41 %) rated their physical health as excellent or very good, with less than a quarter (6, 18 %) describing their physical health as good. By contrast, in the 2011 Canadian Community Health Survey, 69 % of those aged 20 to 34 (the age range of our participants) rated their physical health as excellent or very good. The numbers are even starker for mental health: while 75 % of Canadians consider their mental health as excellent or very good [32] one-quarter (8, 24 %) of participants in our study rated their mental health as excellent or very good [32]. Further, two-thirds (23, 68 %) of participants reported that most days over the last 12 months were “quite a bit” or “extremely” stressful. Participants also reported high rates of diagnosed depression (20, 61 %), and just under half (14, 42 %) reported some degree of long-term disability or handicap. Finally, participants’ scores on post-traumatic stress questionnaires (mean score 48, median 42) was comparable to other vulnerable populations, such as sex workers (40) who face formidable socio-economic challenges and stress, and higher than what is reported for stressful occupations such as first responders, nurses and doctors [33].

We also asked participants how often they had consumed various licit and illicit substances in the past 12 months. The prevalence of past-year alcohol use among participants (22, 67 %) was lower than the prevalence of past-year alcohol use (78 %) reported in the Canadian Alcohol and Drug Use Monitoring Survey (CADUMS), a general population survey of Canadians aged 15 and older (http://www.hc-sc.gc.ca/hc-ps/drugs-drogues/cadums-esccad-eng.php). However, a higher proportion of participations reported past-year tobacco (24, 73 %), cannabis (15, 45 %), and cocaine (9, 27 %) use compared to the Canadian population (17 % tobacco; 10 % cannabis; 1 % cocaine).

While participants had consumed most of the substances we asked about at some point in their lives, three-quarters of them (25, 76 %) also reported having accessed services for drug addiction (e.g., detox, alcohol and drug treatment, Alcohol Anonymous and Narcotics Anonymous). There were negligible differences of reported substance use over the lifetime between women and men interviewed, however, there were large differences between men and women for prevalence during the last 12 months, with women less likely to use most substances.

These differences highlight the influence of gender-based societal and cultural expectations related to substance use, particularly for women and men of childbearing age. One exception to this pattern was that the proportion of women who reported using sedatives in the last 12 months was more than twice that of men (8, 32 % of women; 1, 13 % of men). This mirrors findings from the 2011 Canadian Alcohol and Other Drug Use Monitoring Survey that found significantly higher use of prescription sedatives and tranquilizers among females (12 %) compared to males (6 %) (http://www.hc-sc.gc.ca/hc-ps/drugs-drogues/cadums-esccad-eng.php).

We also assessed participants’ perceptions of substance-related stigma using an adapted version of the *Perceived Devaluation-Discrimination (PDD)* scale [34, 35]. An average score of 5.0 (median 4.9) indicates that participants perceive similar levels of stigma as sex workers, who self-reported 4.8 on the same scale adapted to assess the stigma of sex work [33] and comparatively higher than scores reported in studies of other marginalized populations, including people with mental health conditions [35], people who are legally blind [36], and frontline service providers to sex workers [23].

In summary, compared to the general Canadian population and many other vulnerable groups, the mothers and fathers we interviewed experienced poorer than average levels of physical and mental health, higher levels of stress, depression and disability, and perceived a high level of discrimination toward pregnant and early parenting persons who use substances. These factors constrain opportunities, increase risk, and are linked to negative health behaviours such as substance use. As we report next, the structural vulnerability experienced by participants and the disapproval they feel are interwoven throughout their responses to our research question, “How do you define problematic substance use during pregnancy and early parenting?”

### Discourses on substance use

In contrast to the near universal view of abstinence as the ideal among the service providers we interviewed in an earlier stage of this study, parents were divided on the issue and were also less likely to view abstinence as the ideal and to frame mothers as fetal-vessels [27]. Instead, parents were more likely to place the mother as the centre of the discussion and as the subject of her reproductive experience and to describe her needs as inseparable from the needs of her child(ren). Parents’ responses thus evoked a tension between the socially reinforced and morally laden expectation to abstain from substance use during pregnancy and early parenting and an understanding of the lived experience which allowed for autonomous individuals to judge for themselves what is an acceptable level of substance use. Many mothers and fathers also revealed structural circumstances which they linked to their dependency on substances. Parents believed these structural issues expanded the difficulties in effecting what they saw as desirable lifestyle changes for their own and their family’s health. However, when describing their choices, parents (especially mothers) mostly held a neoliberal view of responsibility for substance use within which they internalized individual shame. Below we provide a table summary of these emergent themes (Table 2 ). In the sections that follow we elaborate on the themes in greater detail.

**Table 2:** Competing discourses in in defining problematic substance use during pregnancy and early parenting

Parallel/competing discourses	
Abstinence (as the ideal)	Autonomy (of individuals to make different choices based on their knowledge and experiences)
Characteristics	
● ‘Obvious’	● Complex
● Normative	● Nuanced
● Deontological/absolutist	● Pluralistic
Informed/reinforced by…	
● Moral conviction	● Relative and contextualized harms
● Traumatic/negative personal experience	● Personal and anecdotal experience
● Ministry policies/legal structures	● Ambiguity of evidence
Sub-themes	
Internalizing a moralized motherhood	A broader view of what influences health
● Delimits the ‘bad mother’ and the ‘good mother’ by substance use and child removal	● ‘Problematic’ determined according to substance type and frequency of use
● Neoliberal view of choice over life circumstances (choice for both substance use and pregnancy)	● Harms mediated by social determinants of health associated with substance use and dependency
● Harm reduction as morally inadequate	● More holistic view of health: personal care, agency, and emotional health
	● Mother and infant health as inseparable
	● Disruption of family as problematic
Results in…	
● Irreconcilable shame and guilt	● Richer discussion of what influences health and child development
● Stigmatization	● Contestation of judgment and stigmatization

#### The competing discourses of abstinence and autonomy

As noted above, participants subscribed, often simultaneously, two competing discourses that inform notions of problematic substance use: the ideal of abstinence and the autonomy of the individual to judge risk for themselves. The former aligned best with the ‘obvious’ mainstream rhetoric [37] and the latter with the nuances of lived experience of and the ambiguity around potential harms. Janet’s definition was typical: “[I]t depends on the substance and how much substance is being used. But, like, obviously abstinence is ideal, for sure”. Janet’s words demonstrate how two competing discourses can become paralleled to form a moral framework that is satisfying to participants: it recognizes individual contexts while upholding the normative ideal.

The normative rhetoric around abstinence during pregnancy and early parenthood is fundamentally moral with motherhood framed as a corruptible bastion of purity and selflessness [37, 38]. Generally, mothers in this study were uncomfortable with deviance from abstinence in what they tended to describe as selfish risk-taking, even when their use was linked with dependency or addiction that pre-dated conception and associated with past traumas. The weight of guilt aligned more closely with the perceived stigma of maternal substance use than with actual evidence of risk to themselves, their partners, or their child(ren). Breaching the moral code of motherhood by doing something “wrong” was often more salient than evidence of harm to a fetus caused by substance use alone. As Margo stated: “I think it’s a problem if you use once. But I used [cocaine], I think three times while I was pregnant, knowing it was wrong, but thought you know, maybe one or two won’t hurt. I was being very selfish”.

Negative or traumatic personal experiences with substances also informed the abstinence discourse for some parents. For example, Kerry, who supported parents’ choice for moderation said: “Nothing would go wrong you know, if I just had a little bit but I just, I can’t see it. I can’t see introducing that type of… opening those doors. Those are dangerous doors to open, in my experience”. Personal experience of addiction led this participant to state that any use may overwhelm the intent to use moderately.

For some participants the parallel discourse of autonomy allowed for moderation according to their personal perception of risks. Melanie regarded alcohol as a less-stigmatized substance and, echoing what she recently read in a government document on drinking during pregnancy, stated that if mothers choose to drink, they should not drink more than one to two units of alcohol once or twice a week” [39].

[T]here’s British standards around drinking are a bit different than the North American standards [in] that they allow for a certain number of units per day, per week actually… So you commonly hear people say abstinence is best around drinking, other people say ‘nope, you can drink a little in moderation’

Pamela believed she was the best judge about what was dangerous and what was permissible for her own health and that of her son:

Like, it’s not excessive or anything. And he’s perfectly fine…There’s a couple of times when I needed a drink to go, help get sleep, you know, just tense and shit. So I made a really, really weak Caesar or something, right.

Many participants similarly stated that other parents were entitled to develop their own perspective on substance use during pregnancy and early parenthood. Even parents who espoused abstinence for themselves had varying levels of tolerance for other parents. As Julianne expressed:

I know some really good mothers out there who will smoke some weed at the end of the night, once their kids are in bed. [L]ike, I don’t agree with it, but at the same time, that doesn’t make them a bad parent.

Notably, parents who did not condemn moderate use only did so for substances that were relatively socially accepted in B.C. (i.e.: tobacco, alcohol, and marijuana). No parent endorsed a moderation model with so-called street or club drugs.

Co-parents were sometimes expected to adhere to the standard of abstinence set by the mothers and were exempt from deciding what level of substance use was acceptable for themselves (further investigation of how mothers versus fathers navigated substance use is included in a separate analysis). As one expectant mother, Michelle, said: “[F]or me, like growing up around alcohol, like, I have no tolerance for it, so I don’t allow myself or my partner to consume alcohol and he’s on board with me on that”.

Finally, for many parents, such as Norah, abstinence was imposed by child welfare agencies enforcing substance use as grounds for child removal: “My thing is that if you drink or smoke or anything, kids are gonna get taken away. That’s what, mostly scared me”.

Overall, the concept of abstinence as the ideal was systematically reinforced by morally laden societal and medical expectations which define any use as unacceptable. While parents, drawing upon their an embodied knowledge of substance use, tended to support individual autonomy and personal choice (in a pluralistic approach that held space for divergent views based on lived experience), the dominant discourse nevertheless moralized any substance use during pregnancy as inherently selfish and wrong.

#### Internalizing a moralized motherhood

Many participants employed a neoliberal view on raising children that focused on the responsibilities of individual mothers, with little recognition of the circumstances within which agency is enacted or even understood. Parents described profound stigmatization around substance use for mothers, especially when children are removed from their care. Sylvia was among the study participants who quit substance use during pregnancy and avoided becoming *that* woman: “You know, I could have picked that path, I could have been that woman who loses her kids to drugs and thank God that’s not me”.

Other mothers interviewed also credited their pregnancies and children with inspiring the motivation to make drastic lifestyle changes. But not all mothers experienced such agency over their circumstances or level of substance use, describing the difficult process of severing ties with partners, friends and family who threatened their ability to discontinue use, even while they depended on these social supports. Several parents saw themselves, as Serena quoted below, as “self-medicating” and spoke of the past traumas that first incited what they viewed as unhealthy substance use; these pre-existing and underlying problems could not be expected to disappear when a couple conceives. According to Serena, who had recently miscarried:

I don’t know, like, just because you’re pregnant it doesn’t magically change what’s going on for you and how you’ve been brought up and all the shit that’s happened to you.

Regardless of their current use or life circumstances, the majority of mothers still reflected a neoliberal view of individual accountability for substance use. As Caroline articulates: “[I]t’s us that have the children inside us and then ultimately it’s our decision. Influences can be blamed, but reality it’s your own, it’s your own doing”. Colleen, who was expecting when interviewed, put it this way:

I think it’s really selfish if you’re gonna use drugs and, continue to be pregnant. I know it’s hard, and not everyone can access services, but, if you really wanna be a mom, and a good mom, you’re gonna do what you have to do.

Part of this neoliberal discourse implied access to abortion services. Some mothers described keeping the pregnancy as a deliberate choice in which they had to gauge their capacity to change their pattern of substance use. Some women chose abortion when personal and structural challenges seemed insurmountable. Sarah explains:

For me, like, like… as using as… say if I had gotten pregnant in 2005, well it was when I didn’t have housing I was very unstable, I was pretty well homeless. Um, and I was heavy in addiction. So if I had been pregnant then, things would have been a lot different. I think I would have had no choice but to get an abortion because, they would just… it would have been impossible to have a baby.

This and similar accounts suggest that women who use substances take many factors into account when deciding to go to full-term, including their health condition, life circumstance, and power to change. Yet weighing the many factors in their lives through a lens of individual responsibility framed the options as abstinence or abortion. The starting point for this process was described by one expectant father, Justin, as “you have to want to be clean”. Jessica, expecting at the time of the interview, agrees:

Problematic is when you’re doing it. You shouldn’t be doing it period, having, being pregnant or having children around. If you’re going to be a substance user or a drug user I would say that’s probably something you should do on your own time. Or take the best effort you can to get away from it, and I would know because it’s hard not doing it and hard not being around it. But it’s all choices.

Most mothers judged themselves for any substance use in pregnancy or early parenting, and expressed their sense of guilt regardless of relative harm or circumstance. The neoliberal perspective espoused by Jessica was self-condemning. She positioned herself within an arduous recovery process, struggling yet determined both to regain custody of her children and to repair her self-image as a mother:

I’m a good person and I know things can change. I just put myself in some bad situations and my children unfortunately are paying for it. And I feel so guilty for that. Overwhelming guilty, maybe that’s why I’m fighting so hard to get them back and prove to myself and them I’m not the mother they make me out to be. (Jessica)

This redemptive state was not always seen as achievable; Haley, who was expecting her third child, was disheartened that the stigmatization did not lift once she felt she had changed:

[I]t’s been very frustrating when people look at you a certain way, and I know I’m repeating myself but, it’s true. And it’s true for everyone in my position, and others too, men too. You get yourself into trouble, you have to pay your dues. People have a hard time letting that go when they see it come up. (Haley)

Thus the attempt to mitigate harms to mother and child or improve life circumstances for families were overshadowed by the perceived continued stigma associated with previous substance use.

#### A broader view of what influences health

While no participant endorsed substance use during pregnancy or early parenting, many defined relative harms according to the dangerousness of the substance and frequency of use. Increasing consumption was connected with progressive health problems for the parent and by extension, the family unit. Service providers’ relative focus on fetal health [27] appeared myopic when contrasted with parents’ broader discussion of a range of personal experiences, factors and causal pathways. Overall and despite the normative medical discourse [37, 38], harms emerged as socially rather than pharmacologically determined. As Kiara, an expectant mother of one, expressed:

Problems with the baby, problems with your household, problems with income. Problems, just, being a drug addict, raising children and having children in that environment. I would think, I don’t know, it’s just not good all-around to have substance problems and try to be a parent. I’ve been there.

Many parents considered substance use to be problematic when it compromised the means required to maintain a healthy home. From this perspective, substance use and health are pitted against each other in a competition for finite material resources [26], which are in short supply for many participants. Some of the service providers and parents converged in their views on this point: substance use that compromises the capacity to meet the family’s basic needs and parental responsibilities is essentially problematic [27]. As one young mother, Janele, stated: “Um, problematic to me would be uh, anything’s problematic, obviously. Um, but where it gets to the point where your bills aren’t paid, where you have no food to eat, where your children aren’t taken care of”. As Janele demonstrated, while substance use may itself pose problems in the family, it is the ensuing poverty that defines what is problematic about substance use.

One of the fathers, Elliot, called attention to the possible strain on family finances but also human resources:

[L]ike where, the uh, the, the money that you’re spending on that is coming out of your, your child’s mouth, I would call that, the problem. The, or… if the money that you’re spending on that, that is coming away from your family that’s one, that’s a financial problem. And if the, the energy that it’s taking from you, is coming out of the time being spent with your baby like if I was, for example, not spending time with my child and, unable to give my family the attention that it needs from that abuse, that’s a problem, right? So, financial and then physical. Yeah. And then emotional, which is, all tied in with the physical.

Elliot further suggested that failing to meet children’s nutritional needs because of poverty may have a greater health effect than moderate alcohol use during pregnancy. In the above account he also describes that parents are usually aware of the self-care necessary for providing physical and emotional support to their children. Nevertheless, pregnancy is also time when realizing good self-care maybe more difficult to manage, especially for mothers, as Elliot states: “your energy levels go down, you’re always tired, it costs more to feed yourself because you want to make sure you get that nutrition”. In Elliott’s nuanced account, the mother, as well as her fetus, was seen as physically more vulnerable during pregnancy; poverty (and the inability to access sufficient nutrition) exacerbated the vulnerability.

Problematic substance use was also constructed from the rhetoric around loss of control or agency that can accompany dependence and addiction. Being ‘high’ was seen as problematic when dis-inhibition or lack of control brought out disturbing or altered behaviour. This is, states Naiva, “when [parents] become a little more loud or, or some… acting silly in the [inaudible] or you like change the behavior around the children, I don’t think that’s good for them to witness”. Overconsumption of alcohol, in particular, was associated with harmful behaviours. Too much substance use, according to Chris, an expectant father, could mire one’s thinking and ability to meet basic needs:

Uh, well, spending more money on drugs than food. Not eating properly. And just so mixed up in drugs it’s hard to, it’s hard to even think sometimes, just ‘cause the addiction’s just got a hold on me. It’s just hard.

Both Naiva and Chris’ accounts share the revelation that it is the way one cares for and relates to their child that can be denigrated by substance use. These observations highlight that social bonding and connectedness are integral to parents’ conceptions of good parenting.

While parents often cited daily use of substances and addiction as key indicators of broader barriers to health, some substances were seen as more problematic than others. Marijuana was described as largely benign, although a chronic state of being under the influence was viewed as incompatible with the attentiveness that parenting requires, and frequent purchases of a substance could jeopardize the finances of an already impoverished home. Although cigarettes were seen as problematic, some parents, like Valarie, smoked to cope with abstention from other substances: “I was just, trying to use the… like less, least harmful substance I could which was smoking” In this way substituting use with substances that were seen as less harmful presented a harm reduction technique. Arden, an expectant mother of one, described the progression of substance type and frequency of use along a spectrum of possible problems:

Problematic? Um… having an addiction, to harmful drugs. So I don’t see pot as being harmful. But, like, if someone was to… I’d say even like, if you were a chain smoker, smoked a lot, I’d say that would be a problem. If you were doing drugs that, caused you to, be with people that could hurt you? Drugs that were, hurting your fetus? That would be a problem. Mm, drugs that made you stop eating for three days, and drinking water, so you’re totally dehydrated or your baby would die, that would be a problem. (Arden)

This participant shows how parents considered the social safety risks as well as potential biomedical risks, and were more likely to see maternal and child safety as intertwined rather than at odds with one another. In this way, the conceptions of parents were more woman (or subject) centred than the conceptions of providers [27]. Parents gave weight to experiences of emotional distress, depression, or anxiety and linked these experiences to their structural backgrounds described above. For example, as the subject of her own experience, Rosemary, an expectant mother of two, pinned the core of problematic to a sense of despair: “Um… my definition would be… when I feel hopeless, like when I feel hopeless I feel like turning to substances. Uh, or […] feeling like substance is the only way to go”.

Most parents did not contest the normative claim that the biomedical pathway for harm to their children was the only one worth considering. However, two mothers explicitly argued that disrupting familial attachments was more harmful for their children than a small amount of exposure to substances in utero. Maggie, an expectant mother of one, recounted the foster parents of her children approaching her with concerns about their behaviour and inquiring about any substance use during pregnancy. Feeling that the marijuana she used during pregnancy had not been harmful, she stated: “They’ve been ripped from every family they’ve known, it’s not about the drugs. […] I’ve done a lot of research and I don’t blame myself for what’s going on and their problems right now other than the fact that I can’t be there for them”.

Overall, parents grounded their discussion in the conditions of their lives, taking into account social support in friends and family, housing and income, and physical and mental health. The degree to which substance use was seen as precluding a healthy life was moderated by type of substance, frequency of use, and any underlying trauma or despair. Expanding on the impact of underlying suffering, Connor, a father of five, described the sense of distress that might pervade a family home because of addiction, despite material and emotional wellbeing:

I always got told and what my grandfather taught me is just, as long as you have a roof over your head and, family that loves you then that’s, that’s a family man. So like, I don’t really know. I disagree for like, if we were alcoholics and everything and, like that and then, that’s not a home. Huh, through my eyes it’s not a home. (Connor)

## Discussion and conclusion

In writing this article, we were interested in the views of vulnerable expectant and new parents directly or indirectly involved with the HerWay Home program regarding substance use during pregnancy and new parenting. The academic literature indicates that substance use as a kind of health behaviour is poorly understood, sometimes being viewed as deviance and disease, and most often viewed as both [40–42]. There is also an intense debate on how to properly conceptualise the origins of substance use behaviour, the conditions under which it occurs and the degree of individual agency users exercise in different social contexts [34, 43, 44]. This is complicated further when we consider the case of substance use during the reproductive period because the pregnant and lactating maternal body is a site where ideological wars are engaged and competing rights claims are generated [19, 45].

Our findings echo themes from other qualitative studies that bring the voices of parents (especially mothers) to the forefront [28, 29, 31]. Parents subscribe to the normative ideals of parenting, especially motherhood, but their accounts contest the stigmatizing notion that parents who use substances do not care about their children [46–49]. Other themes consistent with the existing literature include a nuancing of substance use – i.e., that different substances are associated with different patterns of physiological and social risk to both mothers and children [50]; that harms are embedded in risk environments [26, 48] resulting in multifaceted social disadvantages for parents [46, 51]; and child apprehension is a key concern for parents who use alcohol and illicit substances [17, 46].

In focusing our analysis on conceptions of problematic substance use, we compare the perspectives of parents with those of service providers we studied and suggest implications for practice. In our earlier findings most service providers regarded any substance use during the reproductive period as fundamentally problematic and framed use via a gendered responsibilisation of women as foetal incubators and primary caregivers of infants [27]. The mothers and fathers that we interviewed about the topic were divided, torn between the socially reinforced and morally laden expectation to abstain from substance use during pregnancy and early parenting, and an understanding of the lived experience of substance use that allowed for an individual’s autonomy to judge for themselves what is an acceptable level or not.

For participants endorsing an abstinence perspective, substance use is regarded as essentially harmful, particularly in the context of the reproductive female body, despite the absence of a well-developed body of scientific evidence to support this claim [52]. Even in the context of formidable financial, physical, and emotional hardships, most participants tended to accept full moral responsibility for their substance use without substantially challenging the wider social determinants of poverty, inequity, and marginalization. There was no discussion of resiliency or protective factors that are associated with more privileged upbringings and social status that appear to buffer against the effects of alcohol in utero [53]. Only twice was the potential harm of child apprehension [51] and serial foster care placements compared to the potential harm of substance use in pregnancy.

Professionals who are attuned to our findings of a tension between the parallel discourses of abstinence and autonomy are likely to be more focused and effective in their practices. Critical reflection (and deconstruction) of the deontology of abstinence for expectant and recent mothers (and to a lesser extent, fathers) can provide the space for a more contextualized understanding of client, child, and family health. In this study, parents described the influence of substance use as variable, nuanced and intertwined with the complexities of parenting with limited financial, emotional, and physical resources. Practitioners must also be wary of espousing moral absolutism disguised as utilitarianism when they enact harm reduction and women-centered approaches as means to an end in which the fetal-vessel paradigm continues to prevail. In conducting and developing programs and services, there is a need to privilege the perspectives and leadership of parents who find themselves at the centre of various forms of discrimination. The discord between parents’ and providers’ ideas of problematic substance use shows a need to make space for parents’ to articulate their own needs and understandings of their experiences and material conditions of substance use and parenting.

We also recommend increased attention to the sub-themes of the autonomy discourse: that harms are relative and moderated by many factors, including trauma, poverty, hopelessness, social isolation, level of addiction, as well as substance type and frequency of use [45]. A more holistic perspective of health acknowledges the inseparability of maternal and child health, and recognizes the harms inherent in disrupting early bonding and familial attachment, weighing these against the normative biomedical perspective. Acknowledging the moral frameworks that dominate the public rhetoric around parental substance use without imposing or subscribing to them, may allow providers to support parents’ efforts to create wellbeing within their own individual definitions of problematic – which may prioritize housing and other socioeconomic needs. Most saliently, our study highlights how parents who use substances espouse normative moral discourses as well as a more nuanced perspective in which socioeconomic factors moderate, and to a large extent, determine the harmfulness of substance use.
